# Age-Dependent Utilization of Shelters and Habitat in Two Reptile Species with Contrasting Intraspecific Interactions

**DOI:** 10.3390/ani9110995

**Published:** 2019-11-18

**Authors:** Aleksandra Kolanek, Stanisław Bury, Edyta Turniak, Mariusz Szymanowski

**Affiliations:** 1 Department of Geoinformatics and Cartography, Institute of Geography and Regional Development, University of Wroclaw, pl. Uniwersytecki 1, 50-137 Wrocław, Poland; mariusz.szymanowski@uwr.edu.pl; 2 NATRIX Herpetological Association, ul. Legnicka 65, 54-206 Wrocław, Poland; stanislaw.bury@gmail.com (S.B.); edyta.turniak@gmail.com (E.T.); 3 Institute of Environmental Sciences, Jagiellonian University, ul. Gronostajowa 7, 30-387 Kraków, Poland

**Keywords:** age-dependence, spatial ecology, intraspecific predation, reptiles, habitat use

## Abstract

**Simple Summary:**

Intraspecific interactions are known to affect habitat use in birds and mammals but their role in spatial ecology of reptiles is far less recognized. Our comparative study shows that species known to exhibit intraspecific predation (smooth snake *Coronella austriaca*) express clearly different patterns of habitat and shelter occupancy than a species with no such cannibalistic behavior (slow worm *Anguis fragilis*). Specifically, juvenile smooth snakes prefer sites and shelters not occupied by the adults, even despite suboptimal habitat conditions. We propose that such division indicates an avoidance of predation pressure set by larger individuals on the younger and smaller ones. On the contrary, in slow worms no tendency for intraspecific avoidance are observed, since specimens of different ages commonly share the same area and shelters. This points to higher flexibility in habitat use in slow worms, while the smooth snake population is spatially structured, with juveniles dispersed to the margins of the population range. For endangered smooth snakes, habitat conservation should therefore include a wide buffer zone to maintain the youngest fraction of the population. Future studies on habitat utilization in squamates needs to pay more attention to the social cues, a commonly overlooked aspect in the spatial ecology of reptiles.

**Abstract:**

Reptiles undergo worldwide decline driven mostly by habitat change. Detailed recognition of factors underlying spatial structure and habitat utilization is therefore a prerequisite of effective conservation of this group. While the body of data on spatial ecology of reptiles is rapidly growing, studies on social factors remain still underrepresented. We studied age-specific patterns of shelter use, range size, and habitat preferences in the context of intraspecific interactions in the smooth snake *Coronella austriaca*—known to exhibit intraspecific predation—and the limbless lizard slow worm *Anguis fragilis*—with no such behavior observed. Despite smaller availability of preferred microhabitats, juveniles of smooth snakes occupied habitat and shelters located at the edge of the population range that did not overlap with adults. No such pattern was observed in the slow worm. Our study indicates that intraspecific interactions affect the spatial ecology of squamates. Passive and active protection of habitat must include wide buffers to preserve the poorly detectable young fraction of the population.

## 1. Introduction

Loss of natural habitats and changes in their structure are among the major challenges in biodiversity conservation [1]. Understanding how species use their habitats is therefore helpful to orientate management and planning of protected areas [2]. Most studies on habitat utilization of terrestrial vertebrates have focused on mammals (e.g., [3]) and birds (e.g., [4]), with amphibians [5] and reptiles receiving less attention (e.g., [6]). In snakes, patterns of habitat use are most commonly interpreted through the lens of size-dependent trophic niche partitioning, i.e., ontogenetic differences in diet [7] or, sometimes, by variation in thermoregulatory strategies [8]. However, recent findings suggest that social interaction may be an important, although overlooked, factor in snake ecology [9,10].

Dietary preference is commonly different between adult and juvenile snakes (e.g., [11]), and it might also differ due to sex-specific body size variation [12,13]. Although dietary niche partitioning is important in snakes, not all shifts in space utilization can be explained by variation in dietary niches (e.g., [14]). Studies have shown that the scent of conspecific may attract snakes and affect the direction of their movement [15]. The opposite reaction, the avoidance of conspecifics, is likely to occur in species exhibiting intraspecific antagonistic behaviors such as cannibalism. Intraspecific predation is expected to promote shifts in habitat choice mainly in juveniles, expected to avoid competition with large-sized adults. So far only studies on other squamates, lizards, show such spatial division between juveniles and adults choosing different branches in trees and shrubs in the cannibalistic common chameleons (*Chamaeleo chamaeleon*; [16]). The avoidance behavior is proposed to be a factor of population regulation in snakes [17], but it still remains unknown whether it could be affected by intraspecific predation.

We aimed to investigate patterns of habitat use in the context of age and intraspecific interactions. We compared two sympatric species of reptiles, the smooth snake *Coronella austriaca* and the legless lizard, slow worm *Anguis fragilis*. These two species show similar habitat preferences, comparable body size, and both have a viviparous mode of reproduction. However, the smooth snake is known to exhibit cannibalism [17,18], whereas the legless lizard is a non-territorial species that does not exhibit cannibalism [19]. Since cannibalistic behavior exerts the strongest pressure from adults towards juveniles, we assume the presence of age-dependent shifts in artificial shelter utilization and spatial distribution by those snakes as an indication of adult avoidance by juveniles. We expect adult and juvenile snakes to occupy different shelters (without overlapping) and to exhibit no tendency for spatial clustering, understood as reduced distance between individuals compared to random placements [20]. In contrast, adult and juvenile legless lizards are expected to either co-occur in the same shelter or to show clustered spatial distribution. Next to social cues, microhabitat conditions can also drive spatial distribution, therefore we have additionally controlled for the variation in microhabitat parameters in the studied area. Snake habitat use is generally related to climate, land cover structure, vegetation, and topography [21,22,23,24]. Given the small scale of the study area, we used the potential insolation and vegetation height to measure environmental conditions around each artificial shelter. These two factors (relatively constant from year to year and differentiated in our study area) can potentially strongly affect the choice of living places by the smooth snake, especially because it is a sedentary species, living in open areas, e.g., moors or glades [25,26]. It is likely that environmental effects have a positive correlation with the occurrence of reptiles in places with greater potential insolation and with lower vegetation. Alternatively, juveniles may occupy more overgrown sites to hide from larger individuals, but at the expense of thermoregulatory opportunities.

Concluding, such data could substantially enhance our understanding of the commonly overlooked cues in spatial ecology of squamates and thus could contribute to the habitat conservation regarding species-specific intraspecific relations.

## 2. Materials and Methods

### 2.1. Study Area

**Figure 1:**
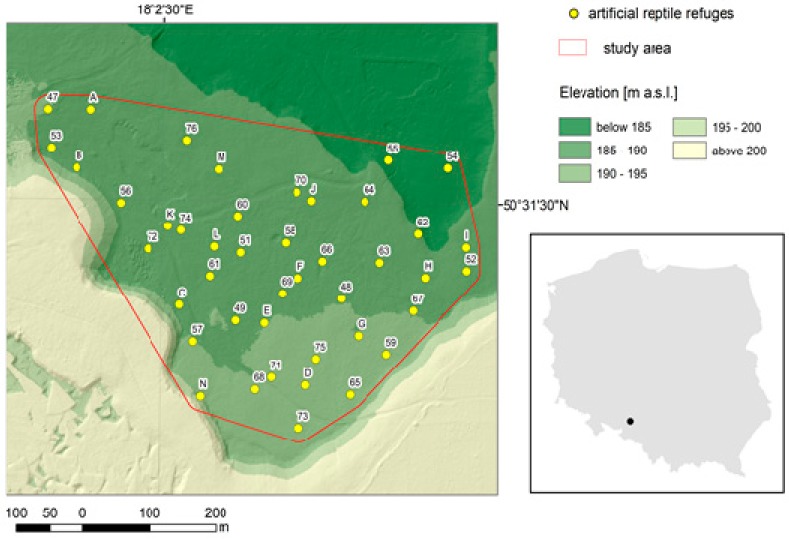
Study area and location of artificial reptile refuges.

The study was carried out in a former limestone quarry located in SW Poland, in Opolskie Voivodeship (Figure 1). The study area is about 21 hectares. It is naturally limited on all sides, from the south, east, and west by steep walls, and from the north by unfavorable habitat conditions—humid areas and active limestone excavation. The area was afforested with pioneer species: pine *Pinus sylvestris* as dominant species and birch *Betula pendula* and alder *Alnus* sp. as admixture. The average age of trees is 20–30 years. The coniferous canopy cover is ca. 32% of the study area, and most of the area lies at elevation from 185 to 190 m a.s.l. The average monthly (Apr–Oct) temperatures were: 9.4, 15.2, 18.3, 19.1, 17.9, 16.0, and 9.2 °C respectively. The spring and autumn were a little bit warmer and the summer was cooler than the long-term average for the nearest meteorological station (Opole), distant approx. 20 km [27]. The amount of rainfall during the research period was 369.55 mm, which was ca 61 mm less than the long-term average sum for the meteorological station in Opole.

### 2.2. Experimental Design and Sampling Data

Artificial reptile refuges (n = 43), made of 1 m × 2 m fragments of roofing felt, were individually numbered and evenly placed in the area (Figure 1). The mean distance between adjacent artificial refuges was 70 m (median = 71 m, range = 21–83 m). Each artificial refuge was surveyed 24 times weekly from April to October 2016. During each visit, we checked for the presence or absence of reptiles on/under the artificial shelters. All specimens observed had their body mass, body length, age, and sex recorded. Body mass was registered using a spring balance (precision = +/− 0.3% of 300 g maximum lifting capacity). We took length measurements three times for each specimen and used the average as body length. We used snout-vent length (SVL) and tail length (TL) in snakes and SVL in lizards, due to the possibility of autotomy. Snakes with more than 400 mm of body length (which is ca. 46% of a smooth snake’s maximum body length reported in Poland, according to Juszczyk [28]) were categorized as adults, whereas smaller specimens were registered as juveniles. Snake sex was determined only for adult specimens based on the thickening of the cloacal area (presence of hemipenes) and on the proportion of tail length to the total length (average 20% in males, 16% in females, according to Juszczyk [28]). In lizards the body length threshold was 140 mm SVL (which is ca. 53% of a slow worm’s maximum body length reported in Poland, according to Juszczyk [28]). The adult lizards were sexed based on the presence or absence of hemipenes. The size-based distinction fits with the data on maturation in both species in southern Poland [28]. Additionally, every smooth snake specimen was marked by ventral scale clipping [29], and the head pattern was photographed. Every slow worm was marked by using pen-like medical cautery units [30].

The base for further analyses was a set of data points containing the geographical location of artificial refuges with information about each individual found, date of observation, morphometry, and information about their age and sex.

### 2.3. Data Analysis

We used the chi-square frequency test to check if the number of artificial shelters occupied by adult snakes, young snakes and both groups is different from the expected number (equal frequencies in all three groups of artificial shelters).

To determine whether adult and juvenile subpopulations of reptiles occupy different ranges, two different methods are available, the minimum convex polygon (MCP) and kernel density estimation (KDE). Both approaches have their drawbacks as well as advantages [31,32,33]. The main advantage of MCP is its simplicity, especially to determine the spatial extent of the population and estimate the boundaries of the area occupied [34]. But it is not free from flaws, for instance, MCP does not consider the probability of settling individual parts of the area [35,36]. Although KDE can be a solution for this later issue, it shows problems with choosing the proper smoothing factor for KDE, which has made its use less preferable over MCP in herpetofauna research [37], a recommendation we followed here. To quantify if any spatial differentiation among reptiles was due to the age group, we used two measures of the central tendency: the mean center and the weighted mean center, to compare the centroids of the subpopulation ranges [38]. The first measure determines the geographical center of the area occupied by the given set of observations (animals), whereas the second metrics weights the geographical center by the number of individuals found in each location. If these centers are close one to another, it means that none of the artificial refuges were occupied by a significantly larger number of individuals than the others. If the weighted means are shifted to one direction, it means that some parts of the area were preferred by more specimens.

We checked the spatial distribution of individuals within their specific age-groups by means of the average nearest neighbor (ANN) method [20]. This method is sensitive to the area extent (subpopulation range), because it is based on the average distance from each focal point (individual) to its neighbors in relation to an average distance calculated for randomly spaced points within the area extent [38]. The ANN statistics were calculated separately for snakes and lizards, since we were interested in mutual spatial relations of individuals of each species within their overall population range. If the average distance between observations is smaller than that expected by chance, the pattern is classified as clustered. If the average distance is greater than the random one, then the spatial pattern is classified as dispersed [20]. In both adults and juveniles of smooth snakes we expected a dispersed distribution due to the risk of intraspecific predation. Conversely, the slow worm, a non-territorial lizard species [19], should be characterized by a cluster or random distribution, because they would not avoid each other.

Environmental data were obtained on the basis of two raster layers covering the study area, provided by the Geodesic and Cartographic Documentation Center: Digital Elevation Model (DEM) and Digital Surface Model (DSM) (both layers with 1 m/px resolution). DEM is a digital record of ground surface (in meters above sea level), and DSM, in the absence of buildings in this study area, reflects the vegetation over the ground or the ground itself in non-vegetated places, similarly expressed in meters above sea level. Based on the differences between DSM and DEM, a new raster layer with information about the vegetation height at each point (raster cell) of the study area was received. Insolation (incoming solar radiation) is a physical term meaning the amount of solar radiation incoming to a unit area of the Earth’s surface [39]. In the ArcGIS software it is counted as the sum of solar energy (Wh m^−2^) for each raster cell (in our case: 1 m^2^ of the ground) for the given period, assuming clear sky conditions (potential insolation) and can be calculated using the Area Solar Radiation tool [40]. This tool does not allow for the impact of cloud cover in calculations, but includes various factors such as terrain (slope inclination, aspect, and hillshading) and astronomical and atmospheric elements [41]. The basis for calculations was DSM.

To check whether the habitat conditions (potential insolation and vegetation height) around refuges differ from those for the entire study area, we used the Student’s *t*-test. For that, we defined three zones: the entire study area (a) and areas around shelters inhabited by juveniles (b) and adult (c) snakes. The size of neighborhood around the inhabited shelter was defined as an area of 10 m radius, based on the diurnal displacement of snakes given by [42]. The environmental conditions were characterized by areal statistics (means, standard deviations, and number of cells) calculated for each environmental variable and each zone separately using the Zonal Statistics tool in ArcGIS [40]. Student’s *t*-test was then applied for each combination of zones: (a) vs (b), (a) vs (c), (b) vs (c).

Due to the binary nature of the reptile-to-shelter information at each visit (1—the reptile was found under artificial refuge during the visit; 0—the reptile was not found), we decided to use the logistic regression method [43] to determine if there is a significant relationship between lizard occurrence on snake presence or absence and vice versa.

Spatial analyses were performed in a projected coordinate system EPSG: 2180 (“PUWG 1992”), and the processing extent was limited to the MCP [20], covering all artificial refuges plus 20 m buffer around. Analyses were performed in ArcGIS 10.2 [44], QGIS 3.0 [45], R 3.4.3 [46], and Python 2.7 [47].

All applicable institutional and/or national guidelines for the care and use of animals were followed (permits from Regional Directorates for Nature Conservation in Opole no. WPN.6401.21.2015.TB and WPN.6401.88.2015.Msz).

## 3. Results

**Figure 2:**
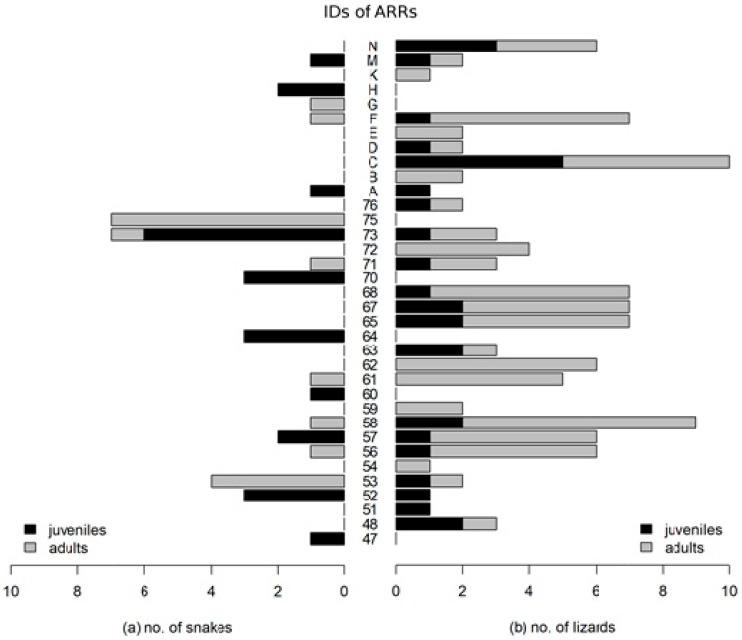
Comparison of the number of adults and juvenile specimens under particular occupied shelters (the IDs according to Figure 1).

During the fieldwork, we observed 43 smooth snakes, including 9 recaptured individuals (number of unique specimens: juveniles n = 17, adults n = 17), and 110 slow worms (60 marked unique specimens: juveniles n = 14, adults n = 46). Snakes were observed under 41.8% (n = 18) of 43 artificial refuges, and juveniles and adults always occupied different shelters with one exception. Slow worms were observed under 67.4% (n = 29) of all artificial refuges. Among those artificial refuges occupied by the slow worms (n = 29), adults and juveniles were found together in 58.6% (n = 17) of the shelters (Figure 2).

**Figure 3:**
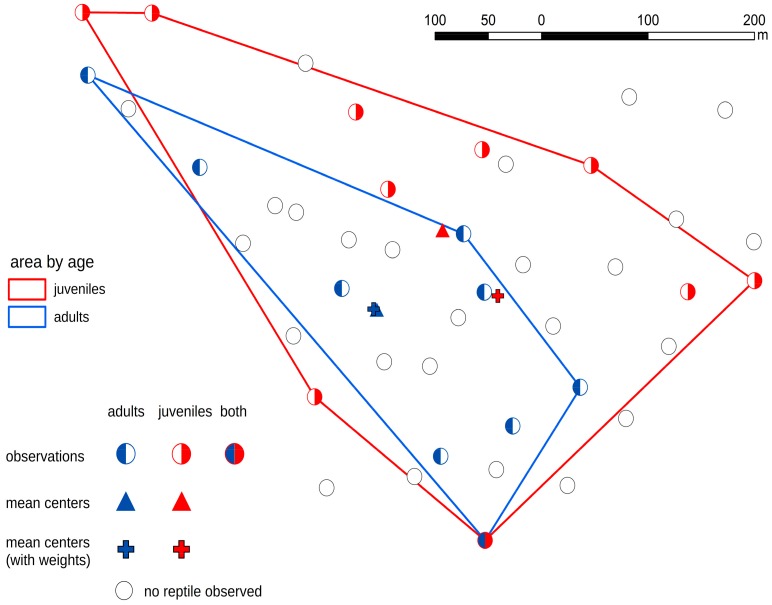
Smooth snakes’ distribution.

**Figure 4:**
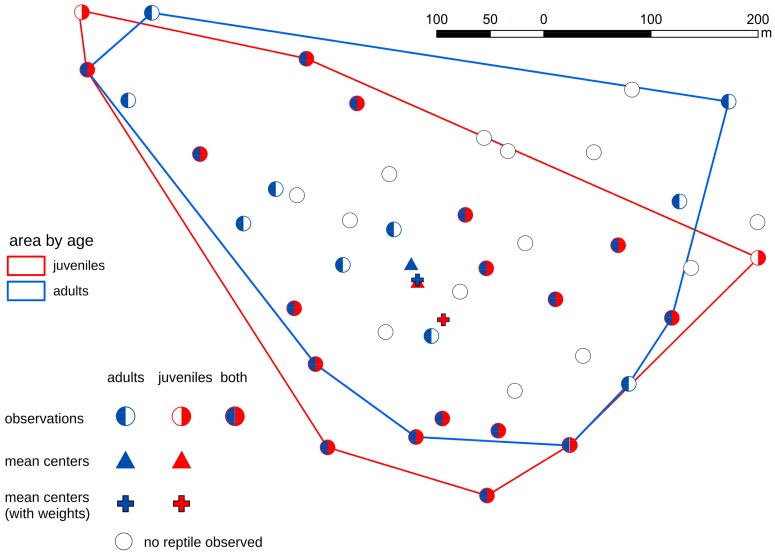
Slow worms’ distribution.

The differences between three groups of artificial refuges (occupied by adults, juveniles, and occupied by both age groups) were statistically significant: for slow worm χ2 = 10.786 (*p*-value = 0.0045, df = 2), for smooth snake χ2 = 6.333 (*p*-value = 0.0421, df = 2). The frequency of shelters occupied simultaneously by snakes of both age groups was lower than the expected, with the opposite pattern in the slow worm. The size of the population range determined by the MCP method was 133,673.6 m^2^ for snakes and 166,187.2 m^2^ for lizards (the whole study area was 212,116.2 m^2^). Spatial distribution of lizards was wider and more evenly spread than that of snakes (Figure 3 and Figure 4).

Lizard specimens showed clustered spatial distribution, except for juveniles, which showed random spatial distribution (Table 1).

Adult lizards were found throughout the area (Figure 4). Weighted centers were shifted to the SE from corresponding mean centers (Figure 4)—43.1 for juveniles, 16.3 for adults. This means that more lizards were observed in the SE part of the area than the NW. The distance between mean centers of the spatial distribution of adult and juvenile lizards was 17.5 m. The size and shape of the population range of both age groups of lizards were similar (MCP of juveniles: 141,710 m^2^, MCP of adults: 166,290 m^2^). Both areas overlapped in 67.9% of study area (Figure 4) and juvenile and adult specimens were found under the same 17 artificial refuges (Figure 2).

The spatial distribution of snake specimens was dispersed, regardless of the age group of the population. Using the MCP method, juvenile smooth snakes occupied a wider area than adults (MCP of juveniles: 128,697 m^2^, MCP of adults: 57,494 m^2^), which occupied only part of the study area (in contrast to adult lizards), and juvenile snakes were not found in the center of adult snakes’ range, only on the edge and outside (Figure 3). Due to that, quantification of overlapped areas is not possible. The shift of the weighted center for the juvenile snakes was 76.3 m to SE from mean center —there were more juveniles in the southeastern part of the study area. Adult snakes occupied their area evenly, so both centers lay almost in the same place (3 m difference). The distance between mean centers (91.4 m) for juvenile and adult snakes was greater than that in lizards and its shift towards the NE expresses differences in the spatial distribution of these two groups (Figure 3).

Juvenile snakes occupied artificial refuges located in areas with lower average potential insolation (14,451.6 Wh m^−2^) and higher average vegetation (1.6 m) than artificial refuges occupied by adults (respectively: 15,603.7 Wh m^−2^ and 1.1 m). The average potential insolation and average vegetation height for the whole study area were, respectively, 13,193.4 Wh m^−2^ and 2.3 m. For both variables there were statistically significant differences between zones (a) vs (b), (a) vs (c), and (b) vs (c) at 0.1 confidence level and with *p*-value < 0.1.

The result of the logistic regression showed no relationship between the occurrence of slow worms and smooth snakes and vice versa (for both situations: Z value = 0.033, *p*-value = 0.0974).

## 4. Discussion

Smooth snake and slow worm—two species different in terms of intraspecific behaviors—revealed different patterns in the use of shelters. In the cannibalistic smooth snake, juveniles generally did not share refugees with adults and choose shelters in areas devoid of adults (Figure 3). Such avoidance behavior was absent for the slow worm, with specimens of both age-groups often sharing artificial shelters (Figure 4). Spatial distribution followed different patterns in both species. In the smooth snake no tendency towards clustering was observed, and the juvenile snakes occupied areas outside the range occupied by the adults (Figure 3), even despite lower availability of microhabitats preferred by the species. In contrast, slow worm specimens tended to cluster and were not spatially divided in terms of their age. The patterns observed in the smooth snake represent the outcome of conspecific avoidance, with no such indications in the slow worm. Overall, our results indicate that behavioral cues are important to shape patterns of habitat utilization in reptiles.

The effect of intraspecific behavior on microhabitat choice has been reported in arboreal lizards [16]. However, in terrestrial flat environments the directions of movements are narrowed to two-dimensional space, which may impose a constraint in the availability of microhabitats. In fact, shelters represent limited resources which access may impact population density and trophic relations in snakes [9,48] and lizards [49] According to our data, reptiles with intraspecific predation may appear more susceptible to such constraints, since avoidance of conspecifics limits the simultaneous use of a shelter by a larger number of specimens and may impose a higher dispersal rate of snakes searching for refugees. On the other hand, in species such as slow worm, with no cannibalistic behavior, shelters may effectively be utilized even by several individuals at the same time. Therefore, those species are more likely to cope and persist in habitats with low availability of refuges.

Secondly, the mode of intraspecific relations may impact patterns of spatial distribution. The larger area occupied by juvenile smooth snakes compared to adults indicates higher movement distances of juvenile snakes [50] which can be a direct outcome of the avoidance of intraspecific predation and, through this, enhanced by searching for vacant shelters. Although most available data suggest adults to be the more migratory fraction of the population in snakes [51,52], our data suggest that this is not to be the case in the smooth snake. In fact, previous studies confirm that juvenile smooth snakes exhibit larger movement distances, reaching up to 700 m [50] compared to adult snakes, for which only around up to 450 m was recorded [53]. Therefore, intraspecific predation may serve as a behavioral mechanism underlying dispersion, which, on the other hand, may increase the mortality risk of juvenile snakes. No such pattern was observed in the slow worm, a species that is more sedentary [54] and rather tends to cluster, which corroborates experimental studies showing a tendency to choose sites with conspecific scent [19]. Although this pattern may reduce mortality associated with movements, it also increases susceptibility towards habitat loss. Such a tendency was already proposed for species representing sit-and-wait foraging strategy [55], and here we propose that it may concern a broader range of sedentary reptile species.

While we have ruled out environmental cues, and our model species seem to rely on intraspecific predation or its lack, one cannot excluded further explanations associated with social behavior that may apply to other species as well. These include mainly territorial behavior and the opposite, i.e., guarding of conspecifics such as offspring, as observed in rattlesnakes [10]. Our study was performed on two reptile species, therefore it is necessary for future research to include a wider array of species that differ in terms of intraspecific behavior to get insight in the global patterns of behaviorally driven patterns of space utilization, mainly in snakes.

Our findings also provide a contribution to reptile conservation. As we have shown, the two species studied by us exhibit entirely different patterns of space utilization, which may have significant consequences for population viability in relation to any changes in habitat structure. In the smooth snake the wider range of area occupied by juveniles than adults indicated the need for planning wide margins of protected areas. This is especially relevant since juvenile smooth snake are more difficult to detect in field surveys which poses the risk of underestimating the area occupied by the population. Moreover, the availability of shelters, commonly underestimated, needs to be taken into account whenever smooth snake habitat is managed. Although slow worm seemed to be more flexible in shelter use and its tendency to clustering may allow it to cope with small habitat patches, it also imposes a higher risk of extinction whenever habitat loss occurs. For both species, as well as many others, we propose the use of geoinformatics along with traditional field surveys. Such a GIS-based approach allows extracting the patterns of habitat use far more precisely than regular surveys and thus may improve habitat-based conservation.

## 5. Conclusions

- Smooth snakes and slow worms showed different age-dependent spatial distribution.
- In the case of the smooth snake, we discovered that juvenile specimens occupied different artificial shelters from adults. In the case of lizards, there were no such dependencies.
- Juvenile snakes chose sites with suboptimal habitat conditions probably because sites with better habitat condition are occupied by adult snakes.
- Taken together, our results provide strong support for the role of age and behavioral cues in shaping the spatial ecology of terrestrial squamates.
- Our findings may also indicate that habitat-based reptile conservation needs to account for the social mode of a given species and associated spatial structuring of the population.

## 6. Licenses

Numbers of licenses to use materials from the Polish Geodesic and Cartographic Documentation Center: DIO.7211.796.2016_PL_N and DIO.DFT.7211.10225.2015_PL_N.

## Figures and Tables

**Table 1:** Average Nearest Neighbor results.

Group	Character of the Distribution	ANN Ratio (*p*-Value)
All snakes	dispersed	1.735 (<0.01)
Adult unique snakes	dispersed	1.440 (<0.05)
Juvenile unique snakes	dispersed	4.305 (<0.01)
All lizards	clustered	0.338 (<0.01)
Adult unique lizards	clustered	0.687 (<0.01)
Juvenile unique lizards	random	0.824 (>0.1)
